# Current and future perspectives for *Helicobacter pylori* treatment and management: From antibiotics to probiotics

**DOI:** 10.3389/fcimb.2022.1042070

**Published:** 2022-11-25

**Authors:** Bing Liang, Yang Yuan, Xiao-Jin Peng, Xin-Lin Liu, Xiao-Kun Hu, Dong-Ming Xing

**Affiliations:** ^1^ Qingdao Cancer Institute, The Affiliated Hospital of Qingdao University, Qingdao, China; ^2^ Intervention Neurosurgery, The Affiliated Hospital of Qingdao University, Qingdao, China; ^3^ School of Life Sciences, Tsinghua University, Beijing, China

**Keywords:** *Helicobacter pylori*, antibiotic resistance, eradication therapy, probiotics, *Limosilactobacillus reuteri* DSM 17648

## Abstract

*Helicobacter pylori* (*H. pylori*) is a Gram-negative anaerobic bacterium that colonizes the human stomach and is the leading cause of gastric diseases such as chronic gastritis and peptic ulcers, as well as the most definite and controllable risk factor for the development of gastric cancer. Currently, the regimen for *H. pylori* eradication has changed from triple to quadruple, the course of treatment has been extended, and the type and dose of antibiotics have been adjusted, with limited improvement in efficacy but gradually increasing side effects and repeated treatment failures in an increasing number of patients. In recent years, probiotics have become one of the most important tools for supporting intestinal health and immunity. Numerous *in vitro* studies, animal studies, and clinical observations have demonstrated that probiotics have the advantage of reducing side effects and increasing eradication rates in adjuvant anti-*H. pylori* therapy and are a valuable supplement to conventional therapy. However, many different types of probiotics are used as adjuncts against *H. pylori*, in various combinations, with different doses and timing, and the quality of clinical studies varies, making it difficult to standardize the results. In this paper, we focus on the risk, status, prevention, control, and treatment of *H. pylori* infection and review international consensus guidelines. We also summarize the available scientific evidence on using *Limosilactobacillus reuteri (L. reuteri)* as a critical probiotic for *H. pylori* treatment and discuss its clinical research and application from an evidence-based perspective.

## Introduction

*Helicobacter pylori* (*H. pylori*) is a Gram-negative obligate anaerobic bacteria colonized in the human stomach. *H. pylori* is the most common infection in the world, infecting around 50% of the world’s population. It causes peptic ulcers and gastric cancer, as well as everyday digestive discomfort. Many cases of *H. pylori* are asymptomatic, but all individuals with *H. pylori* infection have degrees of gastritis, which can progress to severe symptoms over time. Eradication treatment of *H. pylori* infection not only improves the gastrointestinal disease associated with it but also reduces the risk of gastric cancer. The current treatment guideline is to eradicate *H. pylori* using a combination of two antibiotics and a proton pump inhibitor known as triple therapy (Chey et al., 2007; Malfertheiner et al., 2007; Fock et al., 2009). In some cases, a fourth drug, the anti-parasitic compound bismuth, is used, wherein we talk about quadruple therapy. The success of medical eradication ranges from 70% to 95%. The rates, however, have been declining due to increased antibiotic resistance. Furthermore, with the rise of gut microbiome research, the potential side effects of antibiotic therapy (particularly repeated long-term antibiotic use) on gut microecology have gained widespread attention (Mohsen et al., 2020). Medical *H. pylori* therapies cause severe side effects, which reduce treatment compliance. They also drive antibiotic resistance and cannot be taken over the long term. Hence, there is a need for effective natural solutions to control *H. pylori*. Researchers have begun to look for other complementary and alternative therapies to address these concerns and challenges. Probiotics are microorganisms that are beneficial to human health. A large body of basic and clinical research focuses on the different health benefits of probiotics, including their use as an adjunct to *H. pylori* eradication therapy. In a series of *in vitro* and *in vivo* studies, *L. reuteri* DSM 17648 has been shown to specifically bind to *H. pylori* in the gastric environment to form co-polymers that interfere with *H. pylori* adhesion to the gastric mucosa and facilitate its elimination, thereby reducing the *H. pylori* load in the stomach. Clinical trials in multiple countries have shown that *L. reuteri* DSM 17648 reduces *H. pylori* load, improves gastrointestinal discomfort, and reduces the side effects of antibiotic therapy in both adults and pediatric subjects. This article focuses on the current status, risks, and treatment strategies of *H. pylori* infection and reviews the relevant research progress and major consensus guidelines. Current scientific evidence for the use of *L. reuteri* DSM 17648 in treating *H. pylori* is summarized, and its clinical research and application are discussed from an evidence-based perspective.

## The current status and risks of *H. pylori* infection

**Figure 1:**
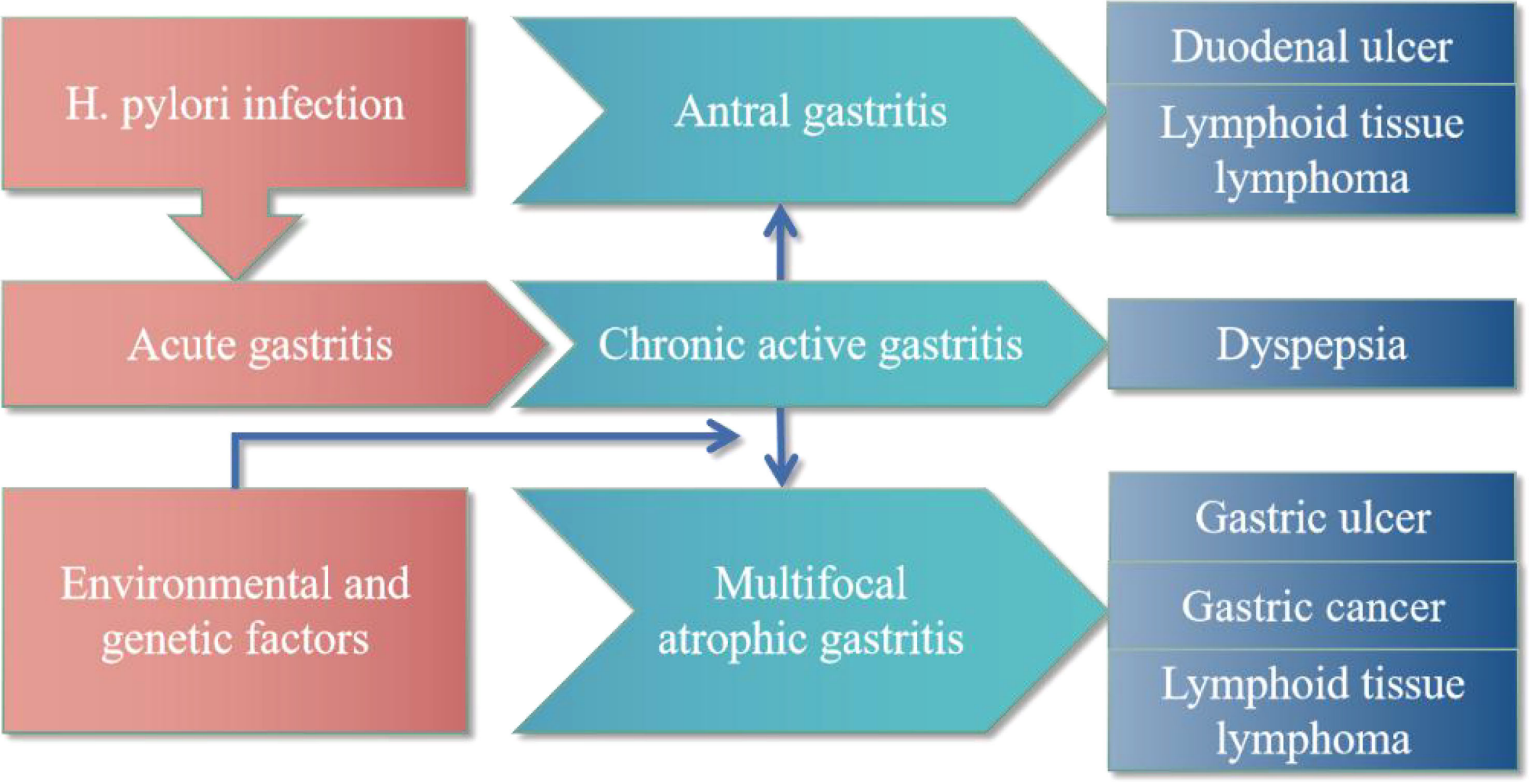
Gastrointestinal disease outcomes of *H. pylori* infection.

*H. pylori* is a common pathogenic bacterium in the stomach and is closely associated with the development of many gastrointestinal diseases (Suerbaum and Michetti, 2002; Malfertheiner et al., 2017) (**Figure 1**). About 25-30% of infected individuals can develop gastrointestinal diseases such as dyspepsia, gastritis, peptic ulcer, and gastric cancer (Liu et al., 2018). The gut microbiota is extremely dynamic and could be influenced by various factors, including host lifestyle, long-term proton pump inhibitor (PPI) use, antibiotic therapy, and *H. pylori* infection (Schmidt et al., 2018; Zhang et al., 2019). Thus, both infection and eradication of *H. pylori* and its interaction with the gut microbiota can alter the microecological balance, thereby affecting the onset and progression of associated diseases (Chen et al., 2022; Fakharian et al., 2022). After infection, *H. pylori* can adapt to the stomach’s harsh acidic environment (Jones et al., 2018; Wen et al., 2018) and interact with host cell receptors to colonize the gastric mucosa (Gonciarz et al., 2019; Hamedi Asl et al., 2019; Saenz et al., 2019), form biofilms (Hathroubi et al., 2018; Ronci et al., 2019), interfere with host metabolic pathways (Hathroubi et al., 2018; Matsunaga et al., 2018), induce neuroimmune crosstalk (Sticlaru et al., 2018), and down-regulate gastric barrier homeostasis (Datta et al., 2018; Liu et al., 2018; Liu et al., 2018; Yaseen and Audette, 2018). More importantly, both *H. pylori* infection and eradication therapy could lead to gut microbiota disturbance, resulting in ecological dysbiosis, promoting gastric carcinogenesis and tumorigenesis (Kienesberger et al., 2016; Ansari and Yamaoka, 2017; Li et al., 2017; Llorca et al., 2017; Guo et al., 2020).

**Figure 2:**
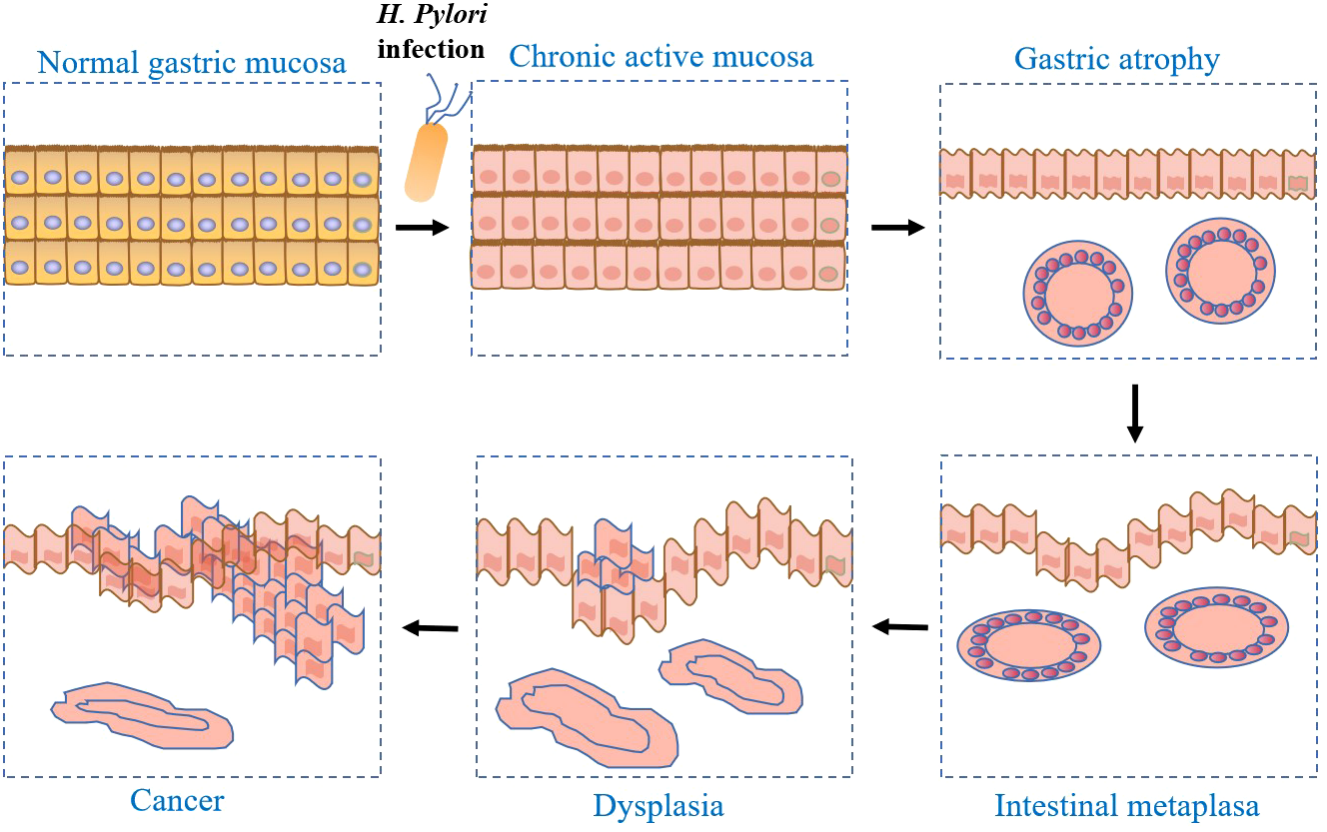
Disease progression pattern of *H. pylori* infection promoting gastric cancer: chronic active gastritis occurs after infection of normal gastric mucosa with *H. pylori*, through progressive development of atrophic gastritis, intestinal chemosis, heterogeneous hyperplasia, and finally gastric cancer.

Fifth Chinese National Consensus Report on the Management of *H. pylori* infection specifically states that peptic ulcers occur in 15-20% of *H. pylori*-infected patients, dyspepsia in 5-10%, and gastric malignancy in about 1% (**Table 1**). Once infected, *H. pylori* infection is difficult to cure on its own without treatment. Due to the high prevalence of *H. pylori* infection worldwide, its related diseases pose a heavy disease burden for human health. *H. pylori* infection is an important cause of gastric cancer (Smyth et al., 2020). Epidemiological surveys have shown that *H. pylori* infection increases the risk of gastric cancer by 4-6 times (Forman et al., 1994). The currently recognized model of gastric cancer disease suggests that *H. pylori* infection drives the progression of the normal gastric mucosa to chronic active gastritis, atrophic gastritis, intestinal metaplasia, dysplasia, and gastric cancer (Correa, 1992; O'connor et al., 2017) (**Figure 2**). Therefore, *H. pylori* eradication is equivalent to the removal of an initiating factor for gastric cancer development. A large meta-analysis also confirmed that *H. pylori* eradication treatment effectively reduced the relative risk of gastric cancer by 46% in healthy infected individuals (Ford et al., 2020). Overall, the prevalence of *H. pylori* infection has decreased in developed and some developing countries, which may be mainly related to improving living standards and hygiene conditions. Notably, the overall global prevalence of *H. pylori* infection is still higher than 40%, and according to the predictions of the meta-analysis, the number of people infected with *H. pylori* worldwide could be as high as 4.4 billion (Hooi et al., 2017). Moreover, there is heterogeneity across studies, with some regional studies unable to express overall prevalence. According to this study, there is an overall global annual recurrence rate of *H. pylori* infection (negative test results after eradication therapy and reappearance of positive *H. pylori* at follow-up) of 4.3%. The annual rate of re-infection (re-infection after eradication) is 3.1%, and the annual re-ignition rate (mainly due to unsuccessful eradication programs) is 2.2%. The recurrence rate is mainly related to the level of economic and social development and health conditions. Thus *H. pylori*-associated diseases continue to pose a significant disease burden on human health.

**Table 1:** Incidence of *H. pylori* infection-associated disease in infected individuals and the proportion attributable to infection.

Gastrointestinal Disease	Incidence in patients with *H. pylori* infection	Proportion of diseases attributed to *H. pylori* infection
Chronic gastritis	˜ 100%	˜ 90%
Peptic ulcer	15 ˜ 20%	70 ˜ 90%
Dyspepsia	5 ˜ 10%	˜ 50%
Gastric cancer	˜ 1%	>85%

## Strategies for prevention, control, and treatment of *H. pylori* infection and challenges

According to the Kyoto Consensus and the Maastricht V Consensus, the causal relationship between *H. pylori* infection and chronic active gastritis is in line with the Koch principle. It is an infectious disease transmitted from person to person (Sugano et al., 2015; Malfertheiner et al., 2017). *H. pylori* transmission necessitates three primary conditions: the source of infection, the route of transmission, and the susceptible population. According to the Kyoto Consensus, all patients with *H. pylori* infection require treatment unless countervailing factors exist (Sugano et al., 2015). According to the Taipei Consensus, the greatest benefit is from *H. pylori* eradication in young adults, which are marital and parenting, and eradicating *H. pylori* in young adults can cure *H. pylori*-associated gastritis, reduce the risk of gastric cancer, and eliminate household transmission (Liou et al., 2020). The second is to cut off the transmission route. *H. pylori* is primarily transmitted orally, including oral-oral transmission, fecal-oral transmission, shared appliance transmission and water source transmission (NCMRCFDD (Shanghai), 2021), with oral-oral transmission through saliva being the most common cause of household cluster infections (chewing and feeding) (Yokota et al., 2015). People of all ages are generally susceptible to *H. pylori*, with infants and children being the most vulnerable, with most patients infected in childhood, especially before the age of 12 (Sugano et al., 2015; Chey et al., 2017).

Presently, international consensus emphasizes the importance of eradication treatment for people infected with *H. pylori*. Drugs for the eradication of *H. pylori* include antibiotics and proton pump inhibitors (PPIs), where antibiotics work to kill *H. pylori* directly, while PPIs work to inhibit gastric acid secretion and raise the pH level in the stomach to create the right environment for the antibiotics to exert their bactericidal effect. A single drug cannot eradicate *H. pylori*, and a combination treatment regimen must be used. The main clinical protocols are triple and quadruple therapy and the resulting sequential, concomitant, and mixed therapies. The leading international consensus opinions on *H. pylori* eradication treatment are the Toronto Consensus on the Treatment of Adult *H. pylori* Infection (Toronto Consensus) (Fallone et al., 2016), American College of Gastroenterology Consensus on the Treatment of *H. pylori* Infection (ACG Consensus) (Chey et al., 2017), Maastricht V Consensus (Malfertheiner et al., 2017) and Fifth Chinese National Consensus (Liu et al., 2018), whose primary treatment regimens are summarized and compared in **Table 2**.

**Table 2:** International consensus opinion on the recommended primary treatment regimen for *H. pylori* infection.

Treatment regimens	The fifth chinese national consensus	Maastricht V consensus	Toronto consensus	ACG consensus
**Clarithromycin-based triple therapy**	not recommended	Recommended for low resistance areas, 10/14 days	Recommended for low resistance areas, 14 days	Recommended for low resistance areas, 14 days
**Non-Bismuth Quadruple therapy**				
Sequential therapy	not recommended	not mentioned	not recommended	recommended, 10/14 days
Concomitant therapy	not recommended	recommended, 10/14 days	recommended, 14 days	recommended, 10/14 days
Mixed therapy	not recommended	Not explicitly recommended	not mentioned	recommended, 14 days
**Bismuth Quadruple therapy**	recommended, 10/14 days	recommended, 10/14 days	recommended, 14 days	recommended, 10/14 days
**High dose therapy**	not mentioned	not recommended	not recommended	Only recommended as remedial treatment, 14 days

### Triple therapy

A combination of one PPI with two antibiotics is a triple therapy. The standard triple regimen based on clarithromycin was established in 1996 (Lind et al., 1996). It can be taken for 7 days with omeprazole, amoxicillin, or omeprazole and metronidazole, with clarithromycin doses of 500 mg and 250 mg, respectively. This regimen was the first-line regimen for *H. pylori* eradication at the time because of its low drug intake, short course of treatment, high efficacy, and low incidence of side effects. Later, scholars expanded the triple regimen with levofloxacin or metronidazole, achieving high eradication rates. However, with the increasing rate of antibiotic resistance of *H. pylori* over the years, the eradication rate of these drug-based triple regimens has been below or well below 80%, and the eradication rate is unsatisfactory even if the regimen is extended to 10 or even 14 days (Malfertheiner et al., 2011; Malfertheiner et al., 2012).

### Bismuth quadruple therapy

The classic bismuth quadruple regimen, which dates back to 1995 and consists of PPI, bismuth, tetracycline, and metronidazole, was established before the clarithromycin triple regimen (Graham and Lee, 2015). As clarithromycin triplet was the first-line regimen at that time, bismuth quadruplet was used only as a remedial treatment. As the rate of *H. pylori* resistance to clarithromycin increased, the efficacy of the clarithromycin triplet regimen declined, and bismuth quadruple therapy was relegated to first-line treatment. Bismuth increases the eradication rate of *H. pylori* resistant strains by 30% to 40% (Dore et al., 2016). Despite the high resistance rates of clarithromycin, metronidazole, and levofloxacin, adding bismuth to triple regimens containing these agents has resulted in satisfactory efficacy (Zhang et al., 2015). The Maastricht V, Toronto, and ACG consensus recommend bismuth quadruple therapy as the first-line treatment option due to the high eradication rate and the fact that bismuth is not easily developed drug resistance and has high safety in short-term application.

### Non-Bismuth Quadruple therapy

Depending on the mode of administration, a non-Bismuth Quadruple regimen can be divided into sequential therapy (PPI + amoxicillin for the first 5 or 7 days and PPI + clarithromycin + metronidazole for the second 5 or 7 days), concomitant therapy (4 drugs for 10 or 14 days), and mixed therapy (same as sequential therapy for the first 5 or 7 days and concomitant therapy for the second 5 or 7 days). Of these regimens, concomitant therapy with 3 antibiotics is the most effective in overcoming antibiotic resistance and therefore has the best relative efficacy but also has a correspondingly higher side effect profile. Because sequential therapies are vulnerable to single resistance to clarithromycin or metronidazole, they have been ruled out for adult treatment by the Maastricht V and Toronto consensus (Fallone et al., 2016; Malfertheiner et al., 2017). When both clarithromycin and metronidazole become resistant, non-bismuth quadruple therapy effectively becomes PPI plus amoxicillin two-component therapy, and eradication rates for sequential, mixed, and concomitant therapy are all reduced (Malfertheiner et al., 2017). Non-bismuth quadruple therapy with clarithromycin and metronidazole is not recommended for empirical treatment in areas with >15% dual resistance of *H. pylori* to clarithromycin and metronidazole, according to the Fifth Chinese National Consensus (Liu et al., 2018).

### Combined Chinese and Western medicine therapy

Commonly used combined Chinese and Western medicine therapies include herbal combination Triple therapy or bismuth quadruple therapy, of which herbal combination triple therapy has the most clinical evidence. Studies have found that certain herbal combination triple therapy has comparable eradication rates to bismuth quadruple therapy at 14 days, while clinical symptom relief is superior to bismuth quadruple therapy (Yao X.J, 2018). Chinese herbal medicine combined with bismuth quadruple therapy has been used relatively rarely because of excessive drug use and minor improvement in the eradication rate. According to the Chinese Expert Consensus on Collaborative Diagnosis and Treatment of Gastritis Caused by Helicobacter pylori in Adults, Chinese herbal medicine can be combined with different aspects of bismuth quadruple remedy treatment to identify symptoms in order to improve the eradication rate of bismuth quadruple remedy treatment (Zhang and Lan, 2020). Patients with obvious GI symptoms can use Traditional Chinese Medicine (TCM) supported by evidence-based medical evidence 2 weeks before the application of bismuth quadruple therapy. Patients without obvious GI symptoms can be treated with TCM supported by evidence-based medical evidence for 2 weeks after bismuth quadruple therapy.

### Other therapies

Quinolones have multiple indications. They are widely used in clinical settings and are cross-resistant to one another, so levofloxacin resistance is widespread except in a few areas. Fifth Chinese National Consensus, Maastricht V Consensus, and Toronto Consensus do not recommend levofloxacin-containing therapy for initial treatment, but it can be used as a remedial treatment option for the failure of first-line therapy (clarithromycin-based) and second-line therapy (classical bismuth quadruple regimen); there is evidence that high-dose PPI and amoxicillin in a duo regimen can achieve high eradication rates, but clinical evidence is scarce and has not been included in mainstream consensus guidelines There is evidence that the addition of certain probiotics to conventional therapies can reduce some of the side effects, but there is a lack of solid evidence to improve eradication rates.

Overall, unless there are clear drug sensitivity test results or evidence of low resistance rates, triple therapy is now not an option as a first-line treatment option. The efficacy of non-bismuth quadruple regimens is vulnerable to single or dual resistance to clarithromycin and metronidazole. Bismuth quadruple therapy, with its high eradication rate and low resistance to bismuth, is recommended by major mainstream consensus opinions as the best option for initial treatment or in areas with >15% clarithromycin resistance.

### Challenges

The eradication rate of conventional *H. pylori* regimens is on the decline globally, and the treatment of *H. pylori* infection faces many challenges, including antibiotic resistance, treatment side effects, patient compliance, and re-infection. Although *H. pylori* antimicrobial resistance varies by geographic region, its prevalence has been increasing over time, resulting in therapy failures and low eradication rates (Flores-Trevino et al., 2018; EaY Tshibangu-Kabamba, 2021). In 2017, *H. pylori* were listed by the World Health Organization as one of the 20 pathogenic bacteria that pose a significant threat to human health due to drug resistance (Tacconelli et al., 2018). In 2018, a meta-analysis showed that the current *H. pylori* resistance rates to clarithromycin, levofloxacin, and metronidazole exceed the Maastricht V consensus recommended threshold for high resistance rates (15%) in most regions worldwide, with dual resistance rates of >15% to clarithromycin and metronidazole in some regions. In contrast, resistance rates to amoxicillin, tetracycline, and furazolidone remain low (Savoldi et al., 2018). High clarithromycin resistance has led to decreasing eradication rates of previous first-line clarithromycin-containing triple therapy (PPI/ranitidine bismuth citrate, clarithromycin, amoxicillin/metronidazole). Studies have shown that the eradication rate of clarithromycin-containing triple therapy has decreased to less than 70% (Agudo et al., 2010). In addition to causing secondary resistance to *H. pylori*, antibiotic therapy may also result in many side effects. In 2021, a study of 22,000 patients showed that approximately 23% of patients experienced at least one *H. pylori* eradication-related side effect, with taste disturbance (7%), diarrhea (7%), nausea (6%), and abdominal pain (3%) being the most common (Nyssen et al., 2021). Furthermore, to design optimized *H. pylori* therapy, clinicians must also consider the importance of gastric cancer regression after *H. pylori* eradication, and pre-treatment susceptibility tests using molecular methods could be performed (Abadi and Yamaoka, 2018).

## Probiotic intervention

**Figure 3:**
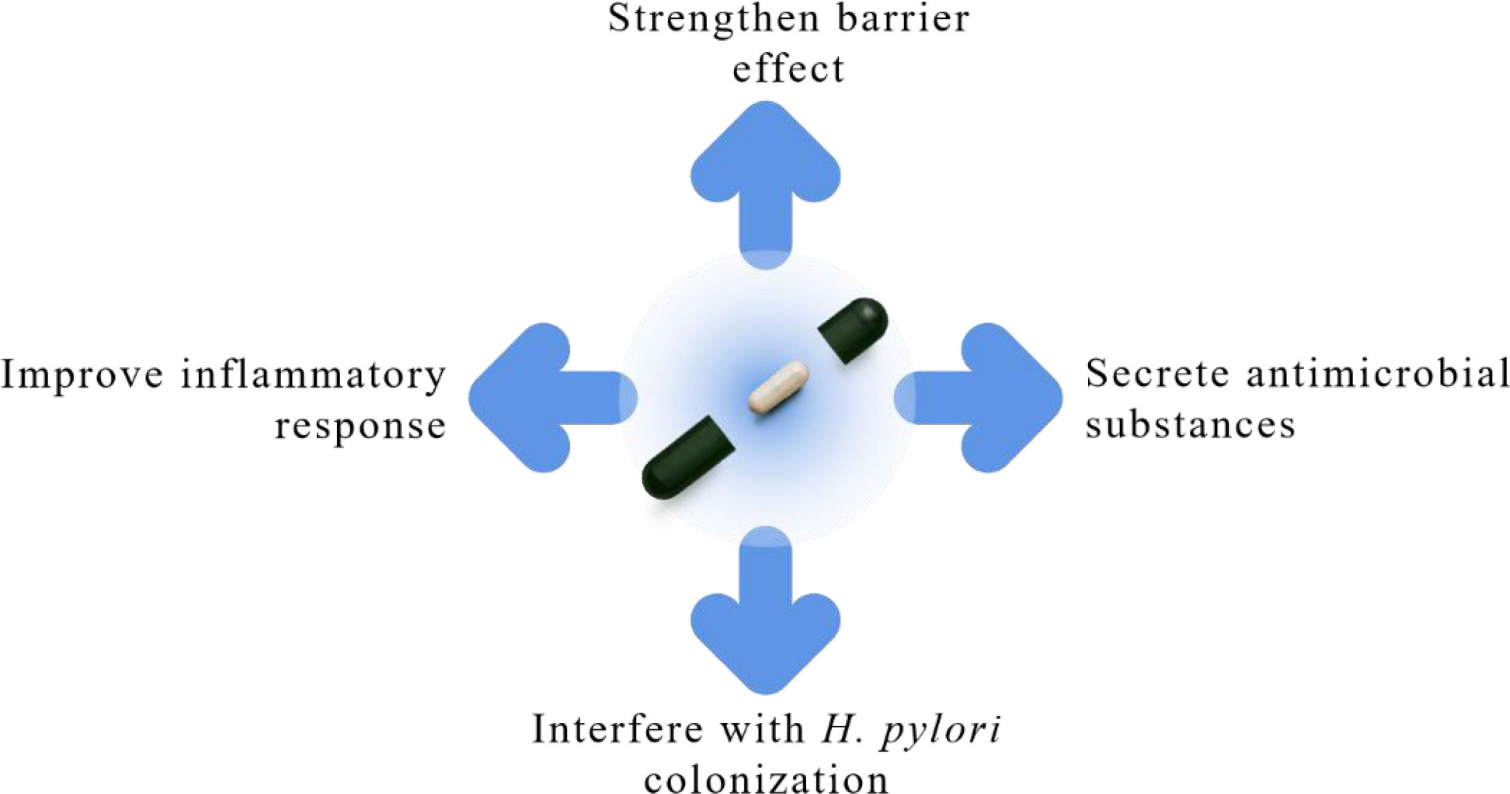
A schematic diagram of the potential mechanism of action of probiotics against *H. pylori* infection.

Due to the decreasing eradication rate of conventional therapies, some studies have begun to focus on the role of probiotics in *H. pylori* eradication. According to the standard definition of the Food and Agriculture Organization of the United Nations and the World Health Organization, probiotics are live microorganisms that, when ingested in sufficient quantities, are beneficial to host health (Hill et al., 2014). Numerous studies have shown that probiotics can benefit the human body in many ways, mainly in improving the health of the gastrointestinal tract (Hill et al., 2014). Current research on probiotics in *H. pylori* eradication treatment focuses on whether the addition of probiotics can improve the eradication rate of *H. pylori*; whether the addition of probiotics can reduce the incidence of side effects and alleviate symptoms in *H. pylori* eradication regimens; and whether the addition of probiotics can promote the restoration of microecological imbalances caused by eradication drugs. The potential mechanisms of action of probiotics to improve *H. pylori* infection include the following aspects (**Figure 3**). First, probiotics may help to enhance the barrier effect (Suez et al., 2019). The gastric acid and mucus barrier of the gastric mucosa are the first line of defense against pathogenic bacteria in the stomach. Some probiotics can upregulate tight junction protein expression, promote mucin and mucus secretion and thus mucus secretion, and enhance the barrier effect of the gastric mucosa. Second, some probiotics can secrete antimicrobial substances, such as lactic acid, short-chain fatty acids (SCFAs), hydrogen peroxide, and bacteriocins (Homan and Orel, 2015). Lactic acid and SCFAs have incomplete dissociation properties, and the undissociated forms of these organic acids can cause damage to *H. pylori*. The anti-*H. pylori* effect of lactic acid and SCFAs is also related to their inhibition of *H. pylori* urease activity. Some probiotics can synthesize hydrogen peroxide and bacteriocins, which also have direct antibacterial effects. Third, probiotics can interfere with *H. pylori* colonization (Qureshi et al., 2019; Ji and Yang, 2020). Some probiotics can interfere with the colonization of *H. pylori* in gastric mucosal epithelial cells by competing for adhesion sites, interfering with the adhesion process, and binding to *H. pylori* to form co-polymers to facilitate its excretion. In addition to these non-immune effects, some probiotics may also reduce the host inflammatory response caused by *H. pylori* infection (Ji and Yang, 2020). The sustained expression of inflammatory factors caused by *H. pylori* infection can lead to a long-term chronic inflammatory response and is an important pathological basis for the pathogenesis of *H. pylori* infection. Probiotics can inhibit the expression of pro-inflammatory factors and improve the inflammatory response in the stomach. Numerous clinical studies have reported the use of probiotics alone or in combination with antibiotics for *H. pylori* eradication. Buckley et al. showed that *L. reuteri* DSM 17648 effectively reduced bacterial load in the stomach and alleviated dyspeptic symptoms in *H. pylori*-infected patients (Buckley et al., 2018). Gotteland et al. showed that using *Saccharomyces boulardii* alone plus inulin resulted in the successful eradication of *H. pylori* in approximately 12% of children (Gotteland et al., 2005), and another study showed that *L. reuteri* combined with PPI therapy resulted in approximately 12.5% eradication of *H. pylori* (Dore et al., 2019). Although probiotics alone have some *H. pylori* eradication rates, they are not a substitute for the bactericidal role of antibiotics in eradication therapy, and supplementing probiotics to an eradication regimen has the potential to improve the effectiveness of antibiotic therapy. **Table 3** summarizes the main results of four recently published meta-analysis studies on the effect of probiotic supplementation on *H. pylori* eradication, suggesting that probiotic supplementation as adjunctive therapy may improve eradication rates and reduce side effects in *H. pylori* eradication regimens (Shi et al., 2019; Yu et al., 2019; Zhou et al., 2019; Zhang et al., 2020). However, some studies have also shown that using probiotics in *H. pylori* eradication therapy is ineffective (Mcnicholl et al., 2018). This may be related to the probiotic supplement, the way and dosage used, the choice of antibiotics for eradication therapy, and the heterogeneity of the included patients. Moreover, diarrhea, sepsis, subacute bacterial endocarditis, and meningitis are all possible side effects of probiotics, but they are scarce (Doron and Snydman. Risk, 2015). Probiotics have potential risks that must be monitored. Thus, much more research is needed to determine the best probiotic and dose for specific diseases, first in animal models comparing different strains, and then in randomized controlled trials (Liu et al., 2018).

**Table 3:** Main results of meta-analysis of the effect of probiotic supplementation on *H. pylori* eradication published in 2019-2020.

Publication date	No. of included studies	Effect of probiotic addition on eradication rate of *H.pylori* (p<0.05)	Effect of probiotic addition on side effects of *H. pylori* eradication (p<0.05)	Reference
Oct-20	40	Increased eradication rate by approximately 10%	Reduced adverse reaction rates by approximately 44%	Zhang et al., 2020
Oct-19	11	Increased eradication rate by approximately 16%	Reduces the incidence of taste disorders by approximately 64%	Yu et al., 2019
Oct-19	18	Increased eradication rate by approximately 9%	Reduced adverse reaction rates by approximately 53%	Zhou et al., 2019
Apr-19	40	Increased eradication rate by approximately 14%	Reduced adverse reaction rates by approximately 53%	Shi et al., 2019

## Scientific evidence of *L. reuteri* DSM 1764 for the relief of *H. pylori* infection

In 2002, a team led by Dr. Christine Lang, a pioneer in German microbiology research, screened more than 700 strains of *Lactobacillus* from its own library *in vitro* and identified a strain with a significant binding effect to *H. pylori*, *L. reuteri* DSM 17648 (Holz et al., 2015). A series of subsequent *in vitro* and *in vivo* studies showed that this strain specifically recognized and bound to the surface protein structure of *H. pylori*, thereby forming a co-polymer with *H. pylori* and excreting it through gastrointestinal motility, thereby reducing the *H. pylori* load in the stomach (Holz et al., 2015). In *in vitro* experiments, the aggregation of *L. reuteri* DSM 17648 with *H. pylori* strains can occur within seconds, and a single *L. reuteri* DSM 17648 cell can bind 2-3 *H. pylori* cells. Moreover, it binds specifically to several different *H. pylori* strains and other species of the genus *Helicobacter*, but not to other gastrointestinal pathogenic bacteria (e.g., *C. jejuni*) and oral and intestinal commensal bacteria (e.g., *E. coli*, *S. salivarius*, *B. fragilis*), suggesting that it should not disturb the normal intestinal flora balance. In addition, *L. reuteri* DSM 17648 effectively polymerizes *H. pylori* in an artificial gastric juice at 37°C. This action occurs in the pH range 2-8 (covering the pH range of gastric juice from fasting to postprandial) and is not interfered with by many common dietary sugar molecules, and requires the presence of pepsin to fully activate this polymerization activity. Unlike probiotics in the usual sense, *L. reuteri* DSM 17648 antagonizes *H. pylori* independently of the bacteria’s own activity - its aggregation activity against *H. pylori* remains effective after inactivation (e.g., spray drying treatment). This property not only reduces the difficulty of transport and storage but also suggests that *L. reuteri* DSM 17648 does not lose its ability to bind *H. pylori* when used in combination with antibiotics, increasing the feasibility of its use in combination with antibiotics.

## Clinical trials of *L. reuteri* DSM 17648 for the treatment of *H. pylori*

The safety and efficacy of the strain have been validated in 12 clinical trials with 951 subjects across 6 countries: Germany, Ireland, Russia, Romania, China, and India (751 subjects involved in published trials so far and 200 subjects in progress) (**Table 4**).

**Table 4:** Completed and ongoing clinical trials with 951 subjects in 6 countries: Germany, Ireland, Russia, Romania, China, and India on the safety and efficacy of *L. reuteri* DSM 17648 strain.

	Country	Study design	Result	Research institutes	Reference
1	Germany	27 subjects; strain safety and efficacy studies	Validate strain screening and testing methods and verify strain safety	HealthTwiST GmbH, Berlin, Germany; Experimental and Clinical Research Center, Berlin, Germany	Holz et al., 2015
		250mg of Pylopass daily for 2 weeks	Significant reduction in the number of *H. pylori* in the body		
2	Germany	22 subjects; strain efficacy study	Significant reduction in the number of *H. pylori* in the body	HealthTwiSt GmbH, Germany	Mehling and Busjahn, 2013
		200mg of Pylopass daily for 2 weeks	Improvement was still observed 6 months after the end of the trial		
3	Russia	30 subjects; strain efficacy study	Significant reduction in the number of *H. pylori* in the body	The Loginov Moscow Clinical Scientific Center, Moscow, Russia	Bordin, 2015
		200mg of Pylopass daily for 4 weeks	Reduced inflammatory infection in 25% of cases; improved indigestion symptoms		
4	Ireland	24 subjects; strain efficacy study	Significant reduction in the number of *H. pylori* in the body	Atlantia Food Clinical Trials, Heron House Offices First Floor, Blackpool Retail Park, Cork T23 R50R, Ireland	Buckley et al., 2018
		200mg of Pylopass daily for 4 weeks	Significant relief of stomach discomfort		
5	Romania	70 subjects; strain efficacy study	The eradication rate was 54.3% in the Pylopass group and 77.1% in the antibiotic group (p = 0.042)	Institute of Gastroenterology and Hepatology, “Grigore T. Popa” University of Medicine and Pharmacy, “Sf. Spiridon” Clinical Hospital, Iaşi, Romania	Mihai, 2019
		Combined triple therapy, 200 mg of Pylopass daily for 4 weeks	Significantly lower side effect rate in the Pylopass group (2.9% vs. 17.1%, p = 0.037)		
6	Romania	46 subjects; strain efficacy study	*H. pylori* eradication rate: 65.22% in the probiotic group and 73.91% in the antibiotic group, with no significant difference between the two groups (p=0.75)	Department of Internal Medicine, Iuliu Hatieganu University of Medicine and Pharmacy, Cluj-Napoca, Romania	Muresan et al., 2019
		Combined triple therapy, 200 mg of Pylopass daily for 4 weeks	In both groups, eradication of *H. pylori* was associated with improved dyspeptic symptoms and anxiety scores in patients, with no significant differences between the two groups		
7	Russia	60 subjects; strain efficacy study	Pylopass in combination with antibiotics increased eradication rate by 10%	Pavlov First Saint Petersburg State Medical University City Clinical Elizabethan Hospital, Saint Petersburg, Russia	Uspienskiy, 2016
		The antibiotic group received pantoprazole for 30 days in combination with amoxicillin and clarithromycin for 14 days; the probiotic group received pantoprazole in combination with probiotics twice daily for 8 weeks. *H. pylori* fecal antigen testing was performed after 30 days of treatment.	Pylopass significantly improves quality of life when used in combination with antibiotics		
8	India	90 subjects; strain efficacy study	Pylopass significantly increased the eradication rate of *H. pylori* and reduced the intensity of gastrointestinal symptoms and treatment-related side effects; the eradication rate of Pylopass combined with triple therapy was 82.69%, significantly higher than the 68.42% in the placebo group	Department of Pharmacy, College of Pharmaceutical Sciences, Dayananda Sagar University, Bengaluru, India	Parth et al., 2021
		Combined triple therapy, 200 mg of Pylopass daily for 2 weeks	*H. pylori* eradication rates were lower in the 60+ age group than in the adult age group		
9	China	200 subjects; strain efficacy study	The eradication rate of Pylopass combined with triple therapy was 86.2%.	Department of Gastroenterology, State Key Laboratory of Organ Failure Research, Guangdong Provincial Key Laboratory of Gastroenterology, Nanfang Hospital, Southern Medical University, Guangzhou, China	Yang et al., 2021
		Combined triple therapy, 200 mg of Pylopass daily for 4 weeks	Significantly lower side effects in the Pylopass group, such as reduced risk of bloating and diarrhea		
10	Russia	49 subjects aged 9-17 years; population-specific efficacy study	*H. pylori* eradication rate of 50%	National Research Tomsk Polytechnic University, Russia	Parolova et al., 2015
		200mg of Pylopass daily for 4 weeks	60% eradication rate when combined with antibiotics, and significant reduction in antibiotic side effects and discomfort		
11	Russia	103 subjects aged 9-17 years; population-specific efficacy study	Pylopass in combination with antibiotics increased eradication rate by 9%	Gastroenterology Department of Saint Petersburg State Pediatric Medical University, Russia	Kornienko et al., 2020
		200 mg of Pylopass twice daily in 2 subgroups for 28 and 56 days in a controlled trial	Significant reduction in ammonia levels in the Pylopass group, demonstrating possible eradication of *H. pylori*		
			Significantly lower side effects in the Pylopass group, such as reduced incidence of diarrhea and abdominal pain		
12	Russia	200 subjects; strain efficacy study	Validation of *H. pylori* eradication rate	N/A	Ongoing
		Combined triple therapy, 200 mg of Pylopass daily for 4 weeks	Validation of the relief of gastrointestinal symptoms in the pre-test, placebo group and test group		

### *L. reuteri* DSM 17648 alone reduces *H. pylori* infection load in adults

Three single-blind, placebo-controlled trials of adult patients infected with *H. pylori* published between 2013 and 2018, enrolling 73 patients, discovered that *L. reuteri* DSM 17648 alone significantly reduced *H. pylori* load in patients and helped reduce mild dyspeptic symptoms with a favorable safety profile (Mehling and Busjahn, 2013; Holz et al., 2015; Buckley et al., 2018). An uncontrolled intervention study of 60 infected adults published in 2016 also showed that *L. reuteri* DSM 17648 preparation reduced *H. pylori* load with a dose-dependent improvement (Bordin, 2015).

### *L. reuteri* DSM 17648 for *H. pylori* eradication in adults, with long-term intervention success rates approaching those of triple therapy and substantially reduced treatment side effects

Two randomized controlled trials published in 2019 (Mihai, 2019; Muresan et al., 2019) enrolled 116 adult patients with *H. pylori* infection with functional dyspepsia and compared the efficacy of *L. reuteri* DSM 17648 formulation in combination with a PPI with conventional antibiotic therapy (triple therapy) for *H. pylori* eradication, antibiotic treatment side effects, and symptom improvement. *L. reuteri* DSM 17648 was found to be less effective than antibiotic therapy in eradicating *H. pylori* in the short term (14 days) and closer to antibiotic therapy in the long term (8 weeks) while having significantly lower side effects and improving symptoms. These results suggest that *L. reuteri* DSM 17648 may be a potential alternative treatment for *H. pylori* infection.

### *L. reuteri* DSM 17648 improves gastrointestinal symptoms and reduces treatment side effects in infected adult with triple therapy

A prospective randomized controlled study reported in 2016 initially explored *L. reuteri* DSM 17648 in combination with triple therapy for treating *H. pylori* infection in 60 patients (Uspienskiy, 2016). This was followed by 2 randomized, double-blind controlled trials published in 2019 and 2021 (Parth et al., 2021; Yang et al., 2021), enrolling 290 adult patients with *H. pylori* infection. It was found that supplementing *L. reuteri* DSM 17648 to standard triple therapy may improve *H. pylori* eradication rates, improve patients’ gastrointestinal symptoms, reduce treatment-related side effects, and protect the intestinal flora. Notably, in both studies, Parth et al. showed that supplementation with *L. reuteri* DSM 17648 increased eradication rates by 20%. In contrast, Yang et al. showed no increase in eradication rates, a difference in results that may be related to differences in patient populations and treatment regimens. The higher eradication rate with triple therapy in Yang et al. may be related to population factors such as a lower mean age (mean age 30 years), which may have masked the effect of *L. reuteri* DSM 17648 to some extent. Further studies are needed to determine the effect of *L. reuteri* DSM 17648 supplementation on triple therapy eradication rates.

### *L. reuteri* DSM 17648 reduces the load of *H. pylori* infection in children, improves symptoms, and reduces the side effects of treatment with quadruple therapy

Two clinical studies on *L. reuteri* DSM 17648 for the adjuvant treatment of children with chronic *H. pylori* infection, reported in 2015 (Ni, 2015) and 2020 (Kornienko et al., 2020), were conducted. A total of 152 children aged 9-17 years with *H. pylori* infection were enrolled in clinical studies of children with gastrointestinal diseases associated with *H. pylori* infection. The study found that long-term treatment (8 weeks) with *L. reuteri* DSM 17648 alone had an eradication rate comparable to that of quadruple therapy for long-term treatment (8 weeks) and improved gastrointestinal clinical symptoms and morphological changes in the gastric mucosa; when used in combination with quadruple therapy, it may increase eradication rates and reduce treatment side effects.

These clinical studies have shown that *L. reuteri* DSM 17648 formulation is safe and effective in reducing *H. pylori* load in adults and children with infection. When used alone, it has a lower eradication rate of *H. pylori* than conventional antibiotic therapy in the short term, but long-term use (e.g., for 8 weeks) may improve eradication rates to near that of antibiotic therapy; in addition, it has significantly fewer side effects and similar improvement in gastrointestinal symptoms compared to antibiotic therapy. When used in combination with antibiotic therapy, *L. reuteri* DSM 17648 has been shown to be effective in improving symptoms and reducing side effects and may further improve eradication rates. However, there are still some inconsistent results in the current studies due to differences in study design, subject populations, treatment regimens, and doses used in different trials. More high-quality, large-scale clinical trials need to be conducted in the future to validate.

Clinical trials have demonstrated that *L. reuteri* DSM 17648 alone can reduce the load of *H. pylori* infection and improve gastrointestinal symptoms in adults and children, while when used in combination with antibiotic therapy, *L. reuteri* DSM 17648 is effective in improving symptoms and adverse reactions in adults and children, and can further improve eradication rates and reduce treatment side effects. According to the findings of this study, *L. reuteri* DSM 17648 could be used as an adjunct to antibiotic therapy in the treatment of *H. pylori* infection and related diseases and may be an alternative therapy for those who are not candidates for antibiotic therapy. Since 2011, *L. reuteri* DSM 17648 and its use against *H. pylori* have applied for and received several patents for inventions granted in Europe, the United States, China, and Japan, as well as intellectual property rights for the trademark Pylopass™. In September 2016, Novozymes, a leading global provider of enzyme and microbial technologies, acquired ownership, patent rights, and a worldwide distribution license for *L. reuteri* DSM 17648 strain. This strain has been evaluated as a food ingredient safe for producing dietary supplements, health foods, and functional foods. Pylopass™ product is a spray-dried powder of *L. reuteri* DSM 17648 with dextrin as an excipient. By the end of 2021, there will be over 110 commercialized products containing Pylopass™ in numerous countries and regions worldwide. Of these, Europe is the most dominant origin with over 50%, and China is the second largest market with over 30 end-brand products.

## Concluding remarks

As a global disease, diseases associated with *H. pylori* infection (gastritis, dyspepsia, peptic ulcer, gastric cancer, etc.) pose a heavy disease burden on human health. Over the last four decades, the prevalence of *H. pylori* infection has gradually declined as economic conditions and public health have improved. However, at least 40% of the population remains infected, with regional variations. Several mainstream international consensus guidelines recommend treating *H. pylori* infection with eradication. However, as *H. pylori* antibiotic resistance grows, the efficacy of conventional antibiotic eradication therapy declines. Recent studies suggest that specific probiotic strains may improve the efficacy of *H. pylori* eradication therapy. *L. reuteri* DSM 17648 (Pylopass™) is a probiotic strain that binds to *H. pylori* in the stomach and forms a copolymer. Several clinical trials have demonstrated that *L. reuteri* DSM 17648 is safe and effective in reducing *H. pylori* load and improving gastrointestinal symptoms in adults and children with the disease; when used in conjunction with antibiotic therapy, it may reduce adverse therapeutic effects and potentially improve eradication rates. However, the current clinical studies have limitations, such as small sample size and unblinded design. More high-quality, large-scale double-blind, randomized controlled trials should be conducted to validate the findings.

Probiotics directly compete with *H. pylori* to help restore the intestinal microbial environment and are more effective than standard triple therapy in treating *H. pylori*-related symptoms. The increasing rate of antibiotic resistance and decreased patient adherence to standard treatment better explain the need for alternative therapies. Adjunctive administration of probiotics to *H. pylori* eradication therapy was associated with higher *H. pylori* eradication rate, reduced diarrhea-related treatment, fewer common side effects, and higher treatment adherence. Thus, although the antagonist activity of probiotics is *H. pylori* strain-specific, with continued and future resistance to antibiotics, probiotics may become a future treatment trend when used alone or in combination with current guideline treatments such as adjuvant therapy, drug delivery systems, and boost of the immune system against *H. pylori* infection.

## Author contributions

LB wrote the manuscript and made the figures and tables. D-MX contributed to the conception of the review. YY, P-XJ, L-XL, and H-XK contributed to the manuscript revision, adjusting figure images and improving the overall language. All authors listed have made a substantial, direct, and intellectual contribution to the work and approved it for publication.

## Funding

This work is financially supported by the Qingdao Postdoctoral Applied Research Project (Grant No. RZ2200001423).

## Conflict of interest

The authors declare that the research was conducted in the absence of any commercial or financial relationships that could be construed as a potential conflict of interest.

## Publisher’s note

All claims expressed in this article are solely those of the authors and do not necessarily represent those of their affiliated organizations, or those of the publisher, the editors and the reviewers. Any product that may be evaluated in this article, or claim that may be made by its manufacturer, is not guaranteed or endorsed by the publisher.
